# Light chain lambda myeloma with fatal AL cardiac amyloidosis in a 21‐year‐old patient: A case report and review

**DOI:** 10.1002/ccr3.2165

**Published:** 2019-05-07

**Authors:** Vincent Camus, Sydney Dubois, Pierre‐Alain Thiébaut, Stéphane Lepretre, Pascal Lenain, Jean‐Michel Picquenot, Elena‐Liana Veresezan, Arnaud François, Dominique Penther, Fabrice Bauer, Arnaud Jaccard, Fabrice Jardin

**Affiliations:** ^1^ Inserm U1245 and Department of Hematology Centre Henri Becquerel and Normandie Univ UNIROUEN Rouen France; ^2^ Department of Internal Medicine Charles Nicolle University Hospital Rouen France; ^3^ Department of Pathology Charles Nicolle University Hospital Rouen France; ^4^ Department of Pathology Centre Henri Becquerel Rouen France; ^5^ Department of Genetic Oncology Centre Henri Becquerel Rouen France; ^6^ Department of Cardiology Charles Nicolle University Hospital Rouen France; ^7^ Centre national de référence de l'amylose AL et des autres maladies par dépôt d'immunoglobulines monoclonales hôpital Dupuytren, CHU de Limoges Limoges cedex France

**Keywords:** cardiac involvement, chemotherapy, daratumumab, light chain myeloma, multi‐organ amyloidosis

## Abstract

Multi‐organ AL amyloidosis is a therapeutic challenge because of light chain deposits severely damaging the function of concerned organs. Cardiac involvement, which leads to concentric hypertrophy of both ventricles, is particularly severe and leads to poor prognosis regardless of combination chemotherapy. This case pinpoints the relevance of combining clinical, histological, and echocardiographic information in the management of this complex and life‐threatening disease.

## 1 INTRODUCTION

We present a unique case of light chain myeloma with multi‐organ AL amyloidosis and t(11;14) in a 21‐year‐old patient. This patient displayed very severe cardiac involvement and died of probable acute heart failure. This case highlights the therapeutic management strategies for this type of extremely rare pathology in young adults.

AL amyloidosis belongs to the group of conformational diseases and is a rare protein disorder, with an incidence of 1 case per 100 000 person‐years in Western countries.^1^ It is associated with the extracellular deposit of free light chains (or, more rarely, heavy chains: AH amyloidosis) of monoclonal immunoglobulins, not cleared by the kidneys and caused by a monoclonal population of B cells. This population is typically a plasma cell population in the bone marrow with low medullary invasion, averaging 7%, but approximately 40% of patients have more than 10% of plasma cells in the bone marrow and therefore hold a diagnosis of multiple myeloma.^2^ The amyloidosis diagnosis is exclusively histological, requires an experienced pathologist, and is established on the finding of amorphous deposits stained by Congo red in optical microscopy and presenting a dichroism and a yellow/green birefringence in polarized light.^3^ Staging of AL amyloidosis is based on the Mayo Clinic Score,^4^, ^5^ using cardiac troponin T (cTnT) or cardiac troponin I (cTnI) and N‐terminal pro‐B‐type natriuretic peptide (NT‐proBNP) dosages with the following thresholds: NT‐proBNP >332 ng/L (or BNP 100 ng/L), cTnT >0.035 μg/L, cTnI >0.1 μg/L, and usTnT >50 ng/L. Depending upon whether NT‐proBNP and troponin levels are both low and high for only one level, or both high, patients are classified into stages I, II, or III, respectively, and in a score modified by European teams IIIA or IIIB with NT‐proBNP lower or higher than 8500 ng/L. This Mayo Clinic Score has been corroborated in multiple datasets, including patients managed with or without stem cell transplantation‐based approaches.^6^ In the absence of adequate treatment, AL amyloidosis has an implacable progressive course due to irrepressible multi‐organ impairment. Patients have a median survival of 1‐2 years, and only 6 months if symptomatic cardiac involvement is present.^7^, ^8^ For the most part, patients are older than 65,^1^, ^8^, ^9^ which complicates treatment management and limits the use of intensive strategies. Clinically pertinent data with relevance to very young patients are sparse.

In this article, we report a unique case of a 21‐year‐old patient diagnosed with lambda light chain myeloma and fatal multi‐organ AL amyloidosis with preponderant cardiac involvement.

## 2 CASE REPORT

A 21‐year‐old male patient, of Senegalese origin, with no relevant medical history, was admitted in January 2018 to the Department of Internal Medicine at Rouen University Hospital for deterioration of the general state, asthenia, weight loss of 18 kg in 8 weeks (20% of his usual weight), and neuropathic lower limb pain. Clinically, the general condition was maintained with a performance status (PS) of 1 and vitals were in the normal range. His BMI was 16, 68. The clinical examination revealed severe undernutrition, orthostatic hypotension, and bilateral neuropathic pain predominating in the right lower limb; the rest of the examination was without abnormalities. The baseline and follow‐up cell blood count, biochemical data, and other important parameters such as NT‐pro‐BNP are provided in Table 1.

**Table 1:** Patient's baseline and follow‐up cell blood count and biochemical data

Date	White blood cell (109/L)	Hemoglobin (g/dL)	Platelet count (109/L)	Creatinine level (µmol/L)	Albumin level (g/L)	NT‐ProBNP (ng/L)	Troponin (cTnT, µg/L)	Serum free lambda light chain (mg/L)	Serum free kappa light chain (mg/L)	Total immunoglobulins (g/L)
January 2018	6.1	14.6	369	94	45	500	0.042	918	7.6	5.9
February 2018	6	14.5	381	82	46	410	0.046	729	7.7	5
March 2018	5.4	12.3	436	88	30	NA	0.158	797	7.8	3
April 2018	7.6	10.9	375	93	29	7823	0.239	689	7.9	2
May 2018	6	11.6	407	114	31	17 214	0.178	745	13.8	2
June 2018	5.5	14.1	432	94	28	11 873	0.252	610	11.5	2
July 2018	4.3	14.6	264	128	26	12 253	0.176	256	6.4	1
August 2018	5.2	10.1	389	115	26	20 174	0.174	118.9	7.3	1

Abbreviation: NA: not available.

Protein electrophoresis found hypogammaglobulinemia at 5.9 g/L. The determination of serum free light chains found a high level of lambda at 918 mg/L, kappa at 7.6 mg/L, ratio at 120, and DFLC = 910.4. The myelogram on a bone marrow aspirate found a reduced cellularity bone marrow (cellularity was estimated at 1.5 on a scale of 0‐4) with rare plasma cells representing 1.5% of the global cellularity. Immunophenotyping by flow cytometry found a very low percentage of plasma cells with a lambda type monoclonal appearance, CD56+ in 2% of plasma cells and loss of CD19 in 79% of plasma cells. Whole‐body bone scan was normal, as it was spinal magnetic resonance imagery (MRI). cTnT was slightly elevated at 0.042 µg/L and NT‐proBNP at 500 ng/L. Holter ECG, diphosphonate cardiac scintigraphy, electromyogram (EMG), and biopsy of the accessory salivary glands revealed no abnormalities. Cardiac MRI revealed diffuse hypertrophy of both ventricles with apex‐predominant hypertrophy of the right ventricle, with preserved left ventricular ejection fraction consistent with diffuse fibrosis.

The patient was then transferred to the Hematology Department of the Henri Becquerel Center in February 2018 due to the suspicion of lambda light chain myeloma with concomitant diffuse amyloidosis.

**Figure 1:**
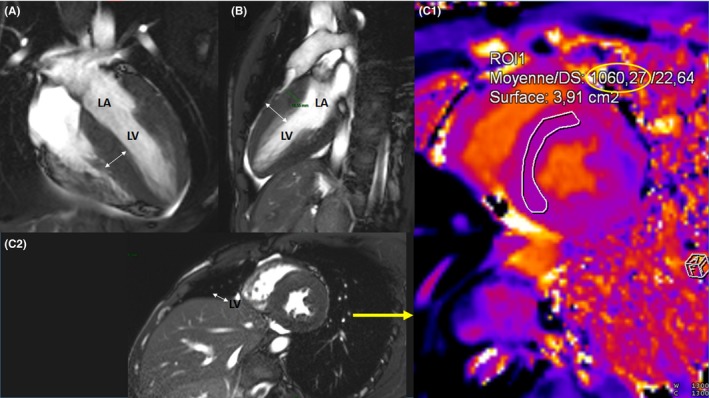
MRI acquisition at baseline showing prolonged T1 mapping consistent with the diagnosis of amyloidosis. (A) and (B) Apical 4‐ and 2‐chamber views by MRI; (C1) Short axis view by MRI; (C2) T1 mapping = 1060 ms (Normal <900 ms); LA, left atrium; LV, left ventricle

Cardiac MRI acquisition at baseline showed prolonged T1 mapping consistent with the diagnosis of amyloidosis (Figure 1).

**Figure 2:**
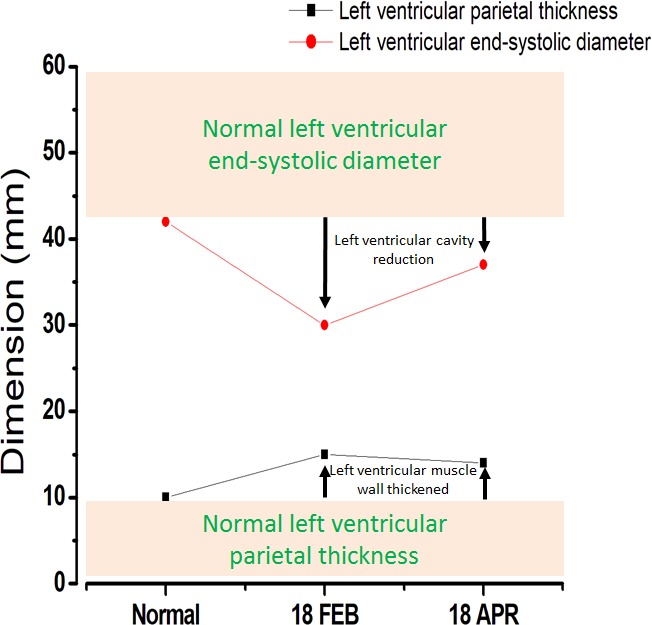
Two serial echocardiographic acquisitions showing thickened left ventricle with concentric hypertrophy

Two serial transthoracic echocardiographic (TTE) acquisitions (Figure 2) demonstrated thickened left ventricle (LV) with concentric hypertrophy. The atria were not dilated. LV hypertrophy was symmetrical with mild medio‐ventricular obstruction, max gradient = 15 mm Hg, without segmental contractility disorder with 63% of LV ejection fraction, without valvulopathy or effusion. Full‐body positron emission tomography (PET) with 18‐F fluorodeoxyglucose was negative. Cerebrospinal fluid (CSF) analysis was normal. The patient temporarily refused myocardial biopsy and then returned to his home at his request.

**Figure 3:**
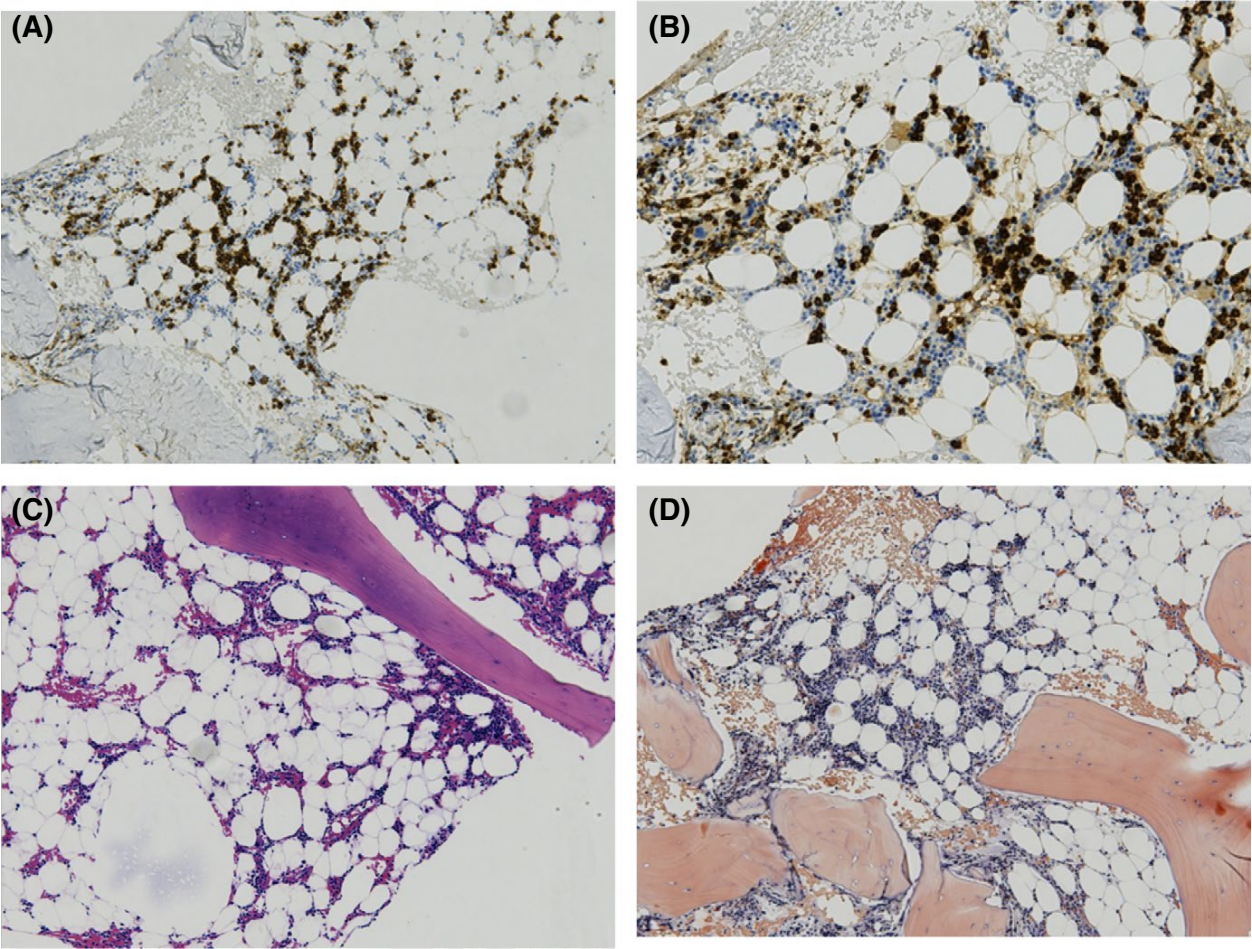
Patient's bone marrow at diagnosis revealing CD138+ massive plasma cell infiltration. Immunohistochemistry staining CD138 (magnification factor ×10). A, Immunohistochemistry staining lambda light chain (magnification factor ×10). B, HES staining (magnification factor ×10). C, Congo red staining (magnification factor ×10)

Due to a worsening of his symptoms (weight loss, severe asthenia, neuropathic lower limb pain), the patient was then readmitted in April 2018 to our Hematology Department. Control of troponin and NT‐proBNP levels increased to 0.239 µg/L and 7823 ng/L, respectively. Bone marrow biopsy showed massive medullary invasion (about 80% of medullary cellularity) by well‐differentiated plasmacytic proliferation with lambda light chain monoclonality, associated with medullary hypoplasia of the three cell lines (Figure 3). The conventional bone marrow karyotype failed twice, but the interphase FISH, on CD138+ sorted cells, found the presence of a double IGH/CCND1 fusion t(11;14) and the loss of an undisturbed IGH copy.

**Figure 4:**
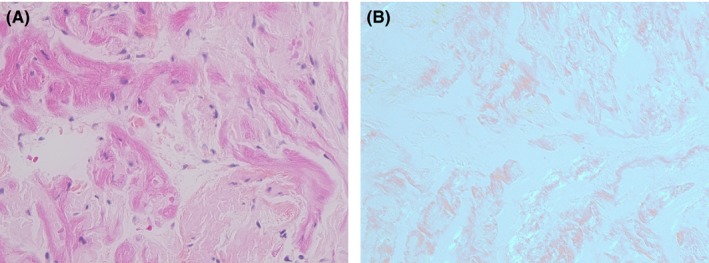
Patient's cardiac biopsy at diagnosis revealing AL amyloidosis. (A) HES staining, magnification factor ×40. (B) Congo red fluorescence assay, magnification factor ×40

Myocardial biopsy of the right ventricle, performed because of the high suspicion of cardiac involvement, confirmed the diagnosis of lambda light chain AL amyloidosis (Figure 4).

We concluded to the diagnosis of lambda light chain myeloma complicated by multi‐organ AL amyloidosis with severe heart involvement (Mayo Clinic stage III) and dysautonomic neuropathy with diarrhea and orthostatic hypotension which was extremely incapacitating. The myeloma CRAB features were as follows: a ratio of lambda/kappa free light chains greater than 100% and 80% of plasma cells on the bone marrow biopsy.

The patient received a first course of treatment with bortezomib (V) (1.3 mg/m^2^ day (D) 1, D4, D8, D11 subcutaneous), lenalidomide (R) (25 mg/d, D1‐D14), and dexamethasone (Dex) (20mg D1‐2, D4‐5, D8‐9, D11‐12) for two 21‐day cycles. The evaluation after C2 showed no therapeutic response (stable disease, IMWG criteria^10^) with lambda light chains at 610 mg/L and kappa light chains at 0 mg/L (DFLC = 610). We proposed a second‐line chemotherapy regimen with two 35‐day cycles of bortezomib 1.3 mg/m^2^ (D1, D8, D15, D22), cyclophosphamide (C) (300 mg/m^2^ D1, D8, D15), dexamethasone (20 mg D1‐2, D8‐9, D15‐16, D22‐23), and daratumumab (16 mg/kg IV weekly) (daratumumab + VCDex regimen). An autologous stem cell transplant procedure with melphalan 200 mg/m^2^ conditioning regimen was considered in case of good therapeutic response and normalization of cardiac markers. The patient presented a biological partial response (PR) with an 80% drop in serum lambda light chain value (lambda light chain 118.9 mg/L, kappa 0.1 mg/L, DFLC = 118.8), and we were able to obtain a collection of peripheral stem cells by two consecutive cytaphereses (under intensive care unit monitoring), which were well tolerated clinically, with a graft of five Million CD34+ cells/kg. Unfortunately, biological PR was not correlated with either clinical response, echocardiographic response (the ventricular hypertrophy was worse, 15‐17 mm against 14 mm at diagnosis), or blood cardiac markers with persistent high levels of troponin (0.174 µg/L) and NT‐proBNP (20174 ng/L). In addition, the patient displayed repeated hypotensive discomfort in connection with dysautonomia, despite midodrine treatment. Given the significant aggravation of weight loss of nearly 5 kg since the beginning of the treatment, the patient underwent nasogastric tube insertion for enteral nutrition to correct severe undernutrition with hypo‐albuminemia at 28 g/L. The patient also presented lower limb edema treated with compression stockings and intravenous 20% albumin supplementation. Finally, the patient was considered not eligible for autologous stem cell transplant because of cTnT >0.06 µg/L and systolic blood pressure <90 mm Hg,^11^ leading to the administration of a third cycle of daratumumab‐VCDex. The patient refused to stay in hospital despite hypotension and major risk of acute heart failure.

Despite all the treatment received, the patient died suddenly at home of probable heart failure, but no medical autopsy was performed. The patient had given his informed consent for the publication of a case report from his clinical history before he died, and we obtained consent to publish this rare case from the patient's next of kin.

## 3 DISCUSSION

We report here for the first time, to our knowledge, the unique case of a very young 21‐year‐old patient, suffering from symptomatic lambda light chain multiple myeloma with multi‐organ AL amyloidosis and t(11;14), resistant to all treatment strategies. The very rapid worsening of the patient's condition in 3 months between the first hospitalization and the specific management in hematology unit is unusual and may be related to the high rate of serum light chains. It should be noted that the association between refractory aggressive myeloma, as in this observation, and amyloidosis is very rare.

Only, a very small proportion (around 2%) of patients with myeloma are diagnosed before the age of 40, and only, 0.3% of patients are diagnosed before the age of 30.^12^, ^13^ It seems that patients younger than 30 years display multiple skeletal lesions with extramedullary localizations and a small monoclonal component with few bone marrow plasma cells^12^ (contrary to our patient's case), but the physiopathology and the characteristics of the occurrence of myeloma in very young patients are not clearly defined. However, a very recent multicenter retrospective study^13^ reported 52 myeloma patients diagnosed between 8 and 30 years of age, with an excellent overall survival rate of 77% at 5 years. Only, 62% of patients had received first‐line treatment with an autologous stem cell transplant (ASCT) and cytogenetic profiles were unfortunately available in less than 50% of these patients, but we can note that the t(11;14) was observed in only 1/20 patients (5%). Our patient presented light chain myeloma; of note, patients younger than 40 years of age have a higher prevalence of light chain myeloma,^14^, ^15^, ^16^ which is possibly a reflection of a different pathophysiological mechanism between younger and older subjects.

Interestingly, the main publications concerning myeloma in young patients (age <40 years), treated before or during the era of novel agents, underline an overall more favorable prognosis than that of classical myeloma patient cohorts.^12^, ^13^, ^15^, ^17^, ^18^

There are also very few reported cases of multi‐organ AL amyloidosis in patients under 30 years of age. Abeykoon et al reported a large cohort of 3433 AL amyloidosis patients evaluated between 1995 and 2015 at Mayo Clinic, and of these, only 52 patients (1.5%) were younger than 40 years at the time of diagnosis.^19^ In this cohort, median age at diagnosis was 38 years (range 26‐40) with data of male sex, lambda, renal, and cardiac involvement preponderance (60, 73%, 65, and 60%, respectively); 12% of patients had concomitant symptomatic myeloma. Of note, FISH techniques were performed in 13 patients and, of these, 69% had t(11;14). This cohort confirms the pejorative prognosis of cardiac involvement (HR 3.9, *P* = 0.004) in this age‐group. No improvement in early mortality has been observed between 1995‐2005 and 2005‐2015 periods.

Regarding treatment management, our patient showed no response to the first‐line treatment (VRD) combining the major drugs used in myeloma.^20^ Second‐line VCD regimen was proposed due to its demonstrated efficacy in high‐risk cardiac AL amyloidosis patients (Mayo Clinic stage III), with 32% of patients displaying a cardiac response and 1‐year OS of 57%.^21^ Nevertheless, because of his refractoriness to the first‐line regimen, we discussed the adjunction of daratumumab (DARA) to this combination, in order to improve the efficacy in this very young patient (DARA‐VCD regimen). A recent report established that high CD38 expression is associated with the presence of t(4;14) and t(11;14) in symptomatic and asymptomatic myeloma, respectively, and is correlated with poor survival in AL amyloidosis, but the biological mechanism of these associations remains undetermined.^22^ It is also known that CD38 expression is not predictive of DARA therapeutic response.^23^ DARA has been recently tested in several retrospective monocentric studies in patients with relapsed/refractory AL amyloidosis. Khouri et al^24^ reported their experience with 20 patients receiving DARA monotherapy and described a strong overall response rate (ORR) of 86% and a short time to hematological response (4 weeks). In a recent Mayo Clinic study,^25^ 22 heavily pretreated patients with AL amyloidosis received a median of eight cycles of DARA‐combined therapy (DARA‐pomalidomide dexamethasone, DARA‐lenalidomide dexamethasone, DARA‐bortezomib dexamethasone) and 22 patients received DARA monotherapy. ORR was 88% in the DARA‐combined group and 78% in the DARA monotherapy group, with a very short time to best response (1.9 months with the combined regimen). Median PFS and OS were not reached in both groups. Notably, the high efficacy of DARA was coupled with a favorable safety profile in both groups. Emerging evidence strongly suggests that DARA, both as monotherapy and in combination, is reliable and highly active in refractory AL amyloidosis. Two prospective phase I/II trials have been conducted in France and in the USA to evaluate DARA monotherapy (NCT02841033, NCT02816476) with very rapid and deep responses in the majority of patients. In our patient, the hematological response to DARA‐based therapy was probably insufficient to drive any clinical benefit, due to the severity of the initial cardiac involvement and the incapacity of this regimen to make disappear light chain deposits on tissues.

Regarding the particular characteristics of our patient, the t(11;14) translocation is known to be much more frequent in AL amyloidosis than in myeloma and generally associated with light chain only AL amyloidosis. There is mounting evidence in the literature supporting the adverse prognosis of t(11;14) translocation in AL amyloidosis,^26^, ^27^ but the pathophysiological mechanism remains uncertain. The aim of the treatment in AL amyloidosis is to decrease the monoclonal protein responsible for deposits through drugs efficient on the clone that synthesizes the amyloidogenic protein.

Another interesting drug in AL amyloidosis is venetoclax, which seems able to induce complete response after VCD in a recent case report^28^ and is currently being assessed in a clinical trial (NCT 03000660). Regardless of the survival increase in recent years thanks to new drugs, there is presently no treatment granting rapid eradication of resident amyloid deposits in tissues.

## 4 CONCLUSION

Light chain multiple myeloma complicated with multi‐organ AL amyloidosis and t(11;14) is a hematological malignancy that can occur in very young patients and is particularly hard to treat. New combinations and clinical trials with molecules such as daratumumab and venetoclax are highly awaited to bring hope for a prolonged CR.

## CONFLICT OF INTEREST

None declared.

## AUTHOR CONTRIBUTION

VC and FJ: involved in the conception and design. VC, PAT, SL, PL, JMP, LV, AF, DP, and FB: contributed to the provision of study materials or patients. VC, SD, FB, AJ, and FJ: involved in the manuscript writing. All authors: involved in the administrative support, collection and assembly of data, data analysis and interpretation, and final approval of manuscript.
